# Disseminated Bocavirus Infection after Stem Cell Transplant

**DOI:** 10.3201/eid1309.070318

**Published:** 2007-09

**Authors:** Thomas Schenk, Brigitte Strahm, Udo Kontny, Markus Hufnagel, Dieter Neumann-Haefelin, Valeria Falcone

**Affiliations:** * Freiburg University Medical Center, Freiburg, Germany

**Keywords:** Human bocavirus, immunosuppression, viremia, respiratory infection, letter

**To the Editor:** Human bocavirus (HBoV) (*1*) is increasingly recognized as a cause of respiratory infections worldwide. Children and infants appear to be most at risk (*2**–**7*), although HBoV’s role in immunocompromised patients remains unclear. We report on a child with disseminated HBoV infection after hematopoietic stem cell transplantation (HSCT). HBoV DNA was detected at high levels in nasopharyngeal aspirates (NPAs) and in blood and stool samples.

A 4.5-year-old boy with dyskeratosis congenita was brought for treatment to our hospital due to severe persistent cytopenia. Allogenic HSCT was performed in August 2006 after conditioning with total body irradiation (200 cGy, day –8 before HSCT surgery), fludarabine (days –7 to –4), antithymocyte globulin (days –4 to –1), and cyclophosphamide (days –3 to –2). He received 7.16 × 10^8^ nucleated bone marrow cells/kg body weight from a 9/10 human leukocyte antigen–matched unrelated donor. Graft-versus-host disease (GvHD) prophylaxis consisted of a short course of methotrexate and cyclosporin A. Neutrophil and platelet engraftment occurred on days 22 and 65 after surgery, respectively. Despite pre- and post-HSCT anti-infective prophylaxis with cotrimoxazole, colistin, acyclovir, and fluconazole, *Enterobacter cloacae* sepsis was diagnosed on day 2. After meropenem treatment, blood cultures remained negative. On day 12, fever reoccurred, elevated C-reactive protein values (229 mg/L) and reduced general health were noted, but no bacterial pathogen was isolated. During this period, the patient received antimicrobial drug therapy with meropenem, tobramycin, vancomycin, and amphotericin B. On day 16, his body temperature peaked to 40.6°C, and a cough and dyspnea without wheezing developed. Chest radiograph results suggested pneumonia with perihilar infiltrates. Reduced oxygen saturation (pO_2_ 86%) was recorded transcutaneously, and oxygen supplementation (maximum 4 L/min) was started by face mask (Appendix Figure). An NPA sample investigated by multiplex PCR (results provided by W. Puppe and J. Weigl; www.pid-ari.net) was negative for adenovirus, respiratory syncytial virus, human metapneumovirus, parainfluenza viruses 1–4, influenza viruses A and B, coronavirus, reovirus, enterovirus, *Clamydia pneumoniae*, *Mycoplasma pneumoniae*, *Bordetella pertussis*, *B. parapertussis*, and *Legionella pneumophila,* but positive for rhinovirus RNA. Retrospectively, the same NPA sample was reanalyzed for HBoV DNA by real-time PCR (*7*) and showed a viral load of 4.6 × 10^7^ copies/mL (Appendix Figure); specificity was confirmed by sequencing.

From day 19 on, the patient’s general health improved and the chest radiograph results returned to normal. After neutrophil engraftment (day 22) and addition of erythromycin to the antimicrobial drug regimen, body temperature decreased and oxygen supplementation was discontinued. However, rhinitis, cough, and low-grade fever (<38.5°C) persisted until day 50 (Appendix Figure), and HBoV DNA was detected in NPAs on days 37 and 44 at 2.4 × 10^11^ and 1.3 × 10^14^ copies/mL, respectively (Appendix Figure). The NPA sample on day 37 was still rhinovirus positive.

Concurrent with the increased HBoV load in NPAs, cytomegalovirus (CMV) reactivation was first diagnosed by PCR on day 20 and peaked (58.250 copies/mL whole blood) on day 41 despite gancyclovir therapy. Switching to foscarnet led to temporary control of CMV replication (Appendix Figure). Additionally, on day 22, acute GvHD grade I with skin manifestations developed. Treatment with steroids until day 60 led to complete resolution.

HBoV infection in this patient was not restricted to the respiratory tract. Diarrheic stool samples obtained on day 21 and, after resolution of respiratory symptoms, on day 75 showed substantial HBoV DNA (2.5 × 10^6^ and 6.0 × 10^5^ copies/mg, respectively; Appendix Figure). Tests for rotavirus and adenovirus antigens were negative, and no bacterial pathogen was isolated. Moreover, HBoV DNA was detected at lower levels (3.7 × 10^3^ to 7.8 × 10^4^ copies/mL) in 4 EDTA plasma samples taken days 21–47. Subsequent plasma (days 61, 68, 75, 88, 219), NPA (day 219), and stool (day 219) samples were negative for HBoV DNA. However, the ability of HBoV to cause persistent infection, as do other members of the *Parvovirinae* subfamily, cannot be excluded. Future investigations are needed to address this hypothesis.

Here, we report on disseminated HBoV infection in an immunocompromised patient. Whether the clinical course in this case was more severe or prolonged than it would have been for HBoV infections in non-HSCT children remains unknown due to the lack of long-term observations in immunocompetent children. The dramatic increase of HBoV load in NPAs and viral dissemination most likely resulted from progressive impairment of cellular immunity as indicated by simultaneous CMV reactivation. Moreover, the increased viral load might have also been a consequence of steroid addition to immunosuppressive therapy to control GvHD. The contribution of HBoV to respiratory disease remains ambiguous because 2 NPA samples were also rhinovirus positive. Additional studies are required to investigate the pathogenic role of HBoV in double or multiple infections. Association of HBoV with the patient’s continued diarrhea is in accordance with previous studies (*8*–*10*). Prolonged fecal shedding has important implications for isolation measures in transplantation units. More studies in immunocompromised patients are required to evaluate the spectrum of pathology caused by this emerging virus.

## Supplementary Material

Appendix Figure Timeline of clinical and virologic features posttransplantation. A) Main clinical events and therapeutic measures. B) Human bocavirus (HBoV) DNA load measured by real-time PCR; 1 mL stool suspension corresponds to 20 mg starting material. Nasopharyngeal samples (NPS) also positive for rhinovirus (RV) RNA by multiplex PCR are indicated by arrows. C) Copies of cytomegalovirus (CMV) DNA measured in blood by real-time PCR. NPSC3.1 plasmid (positive control for PCR) was provided by T. Allender.

## References

[1] Allander T, Tammi MT, Eriksson M, Bjerkner A, Tiveljung-Lindell A, Andersson B. Cloning of a human parvovirus by molecular screening of respiratory tract samples. Proc Natl Acad Sci U S A. 2005;102:12891–6. 10.1073/pnas.0504666102 16118271 PMC1200281

[2] McIntosh K. Human bocavirus: developing evidence for pathogenicity. J Infect Dis. 2006;194:1197–9. 10.1086/508228 17041844 PMC7109811

[3] Ma X, Endo R, Ishiguro N, Ebihara T, Ishiko H, Ariga T, Detection of human bocavirus in Japanese children with lower respiratory tract infections. J Clin Microbiol. 2006;44:1132–4. 10.1128/JCM.44.3.1132-1134.2006 16517912 PMC1393160

[4] Sloots TP, McErlean P, Speicher DJ, Arden KE, Nissen MD, Mackay IM. Evidence of human coronavirus HKU1 and human bocavirus in Australian children. J Clin Virol. 2006;35:99–102. 10.1016/j.jcv.2005.09.008 16257260 PMC7108338

[5] Smuts H, Hardie D. Human bocavirus in hospitalized children, South Africa. Emerg Infect Dis. 2006;12:1457–8. 17073104 10.3201/eid1209.051616 PMC3294732

[6] Weissbrich B, Neske F, Schubert J, Tollmann F, Blath K, Blessing K, Frequent detection of bocavirus DNA in German children with respiratory tract infections. BMC Infect Dis. 2006;6:109. 10.1186/1471-2334-6-109 16834781 PMC1550408

[7] Schenk T, Huck B, Forster J, Berner R, Neumann-Haefelin D, Falcone V. Human bocavirus DNA detected by quantitative real-time PCR in two children hospitalized for lower respiratory tract infection. Eur J Clin Microbiol Infect Dis. 2007;26:147–9. 10.1007/s10096-006-0244-6 17216422 PMC7087733

[8] Kesebir D, Vazquez M, Weibel C, Shapiro ED, Ferguson D, Landry ML, Human bocavirus infection in young children in the United States: molecular epidemiological profile and clinical characteristics of a newly emerging respiratory virus. J Infect Dis. 2006;194:1276–82. 10.1086/508213 17041854 PMC7204143

[9] Manning A, Russell V, Eastick K, Leadbetter GH, Hallam N, Templeton K, Epidemiological profile and clinical associations of human bocavirus and other human parvoviruses. J Infect Dis. 2006;194:1283–90. 10.1086/508219 17041855 PMC7199845

[10] Arnold JC, Singh KK, Spector SA, Sawyer MH. Human bocavirus: prevalence and clinical spectrum at a children's hospital. Clin Infect Dis. 2006;43:283–8. 10.1086/505399 16804840 PMC7107867

