# Supplementary figures and images for: Disseminated Bocavirus Infection after Stem Cell Transplant
Source: Emerg Infect Dis. 2007 Sep;13(9):1425–7. doi: 10.3201/eid1309.070318 (PMC2857292; doi:10.3201/eid1309.070318)

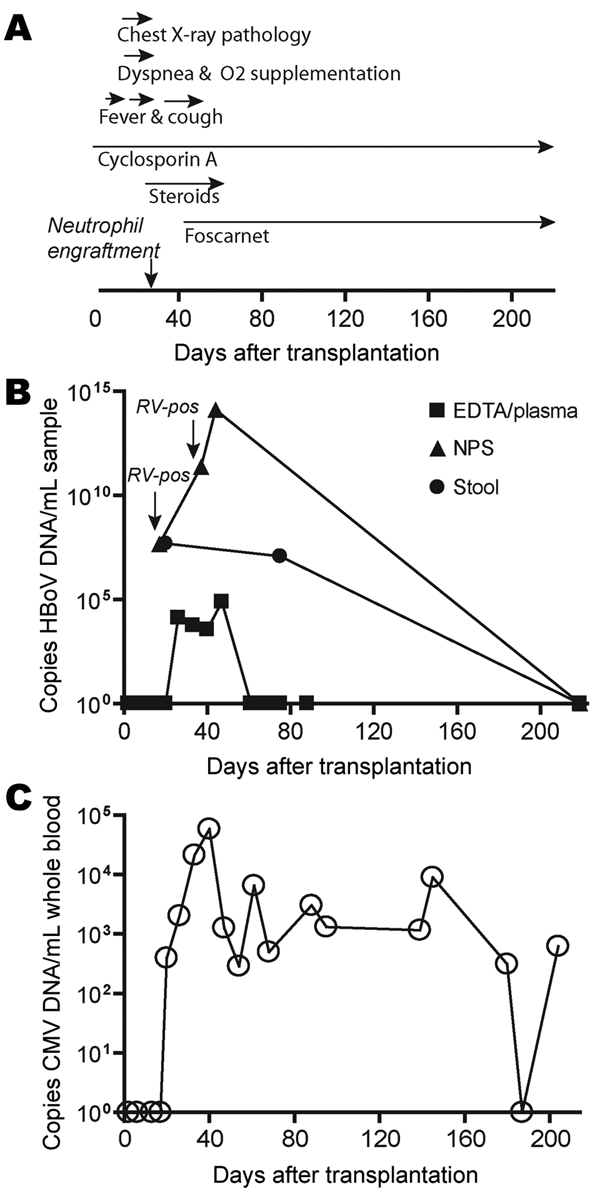

Supplement: Appendix Figure — Timeline of clinical and virologic features posttransplantation. A) Main clinical events and therapeutic measures. B) Human bocavirus (HBoV) DNA load measured by real-time PCR; 1 mL stool suspension corresponds to 20 mg starting material. Nasopharyngeal samples (NPS) also positive for rhinovirus (RV) RNA by multiplex PCR are indicated by arrows. C) Copies of cytomegalovirus (CMV) DNA measured in blood by real-time PCR. NPSC3.1 plasmid (positive control for PCR) was provided by T. Allender. [file 07-0318_appF-s1.gif]
